# Supplementary material for: Implementing Defects for Ratiometric Luminescence Thermometry
Source: Nanomaterials (Basel). 2020 Jul 8;10(7):1333. doi: 10.3390/nano10071333 (PMC7407274; doi:10.3390/nano10071333)
Supplement: Supplementary file 1 [file nanomaterials-10-01333-s001.pdf]

Supporting Information

Implementing defects for ratiometric luminescence thermometry

Joanna Drabik[1*], Karolina Ledwa[1], Lukasz Marciniak[1*]

[1] Institute of Low Temperature and Structure Research, Polish Academy of Sciences, Okólna 2, 50-422 Wroclaw, Poland

* Correspondence: j.drabik@intibs.pl; l.marciniak@intibs.pl

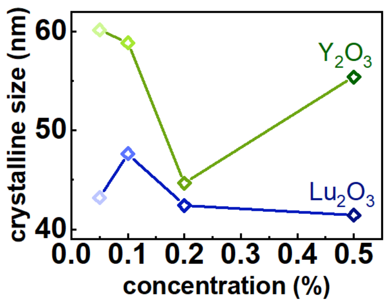

Figure S1. Calculated crystalline sizes of the RE oxides doped with different Tb^{3+} concentrations.

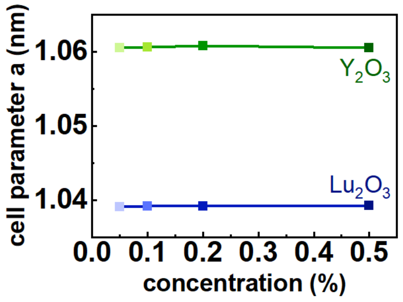

Figure S2. Calculated cell parameter a for the RE oxides doped with different Tb^{3+} concentrations.

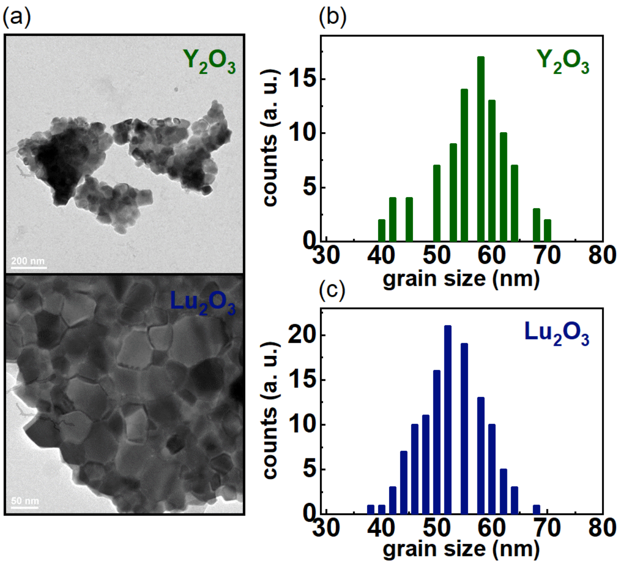

Figure S3. (**a**) Transmission Electron Microscopy images of the nanocrystalline RE oxides doped with 0.5% Tb^{3+} ions (the appropriate scale bar is presented in each image); (**b**) Size distribution for Y_2O_3:0.5%Tb^{3+} and **(c)** for Lu_2O_3:0.5%Tb^{3+}.

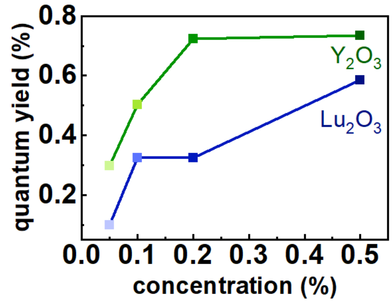

Figure S4. Quantum yield (λ_{exc}=266 nm) for the RE oxides doped with different Tb^{3+} concentrations.

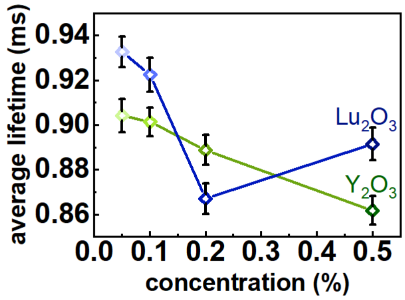

Figure S5. Average lifetimes determined from the luminescence decay curves for the RE oxides doped with different Tb^{3+} concentrations.

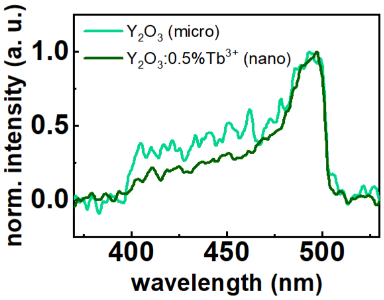

Figure S6. Comparison of emission spectra (λ_{exc} = 543 nm) of microcrystalline precursor Y_2O_3 (of 99.999% purity from Stanford Materials Corporation) with synthesized nanocrystalline Y_2O_3:0.5%Tb^{3+} at the same temperature.
